# Fournier’s Gangrene in the Setting of Underlying Carcinoma: A Case Report and Review of the Literature

**DOI:** 10.7759/cureus.10317

**Published:** 2020-09-08

**Authors:** Adam J Mann, Dixie B Reinoso, Thomas Genuit, Jesus Jimenez

**Affiliations:** 1 General Surgery, Florida Atlantic University Charles E. Schmidt College of Medicine, Boca Raton, USA; 2 Neurology, Cleveland Clinic Florida, Weston, USA

**Keywords:** fournier's gangrene, gastrointestinal oncology, uro oncology, colorectal cancer, sarcoma soft tissue

## Abstract

Most case series of Fournier's gangrene (FG) do not list malignancy as a cause; however, isolated cases with underlying malignancy of the soft tissue, genitourinary, and gastrointestinal systems have been described. After a review of recently published literature, 20 case reports and 15 case series or review articles included relevant information and were included in this literature review.

Malignancy is overlooked in 10% (2/20) of patients, resulting in a delayed diagnosis and initiation of cancer treatment. All patients with FG should have a thorough cancer history, digital rectal examination, appropriate local and systemic imaging, as well as tissue biopsies, to reduce the likelihood of a missed cancer diagnosis. Delay in management of the local malignancy may lead to persistence or recurrence of the infection and significantly worsens overall outcome and survival.

## Introduction

Fournier’s gangrene (FG) is a rare and often severe necrotizing soft tissue infection of the perineum and genital region. A primary etiology can be identified in up to 95% of cases, including folliculitis, hidradenitis and perirectal/perianal abscess, invasive anorectal or urogenital procedures (i.e., hemorrhoidectomy, treatment of urethral stricture, indwelling catheters), and penile intravenous drug use [1]. Most case series of FG do not list malignancy as a cause, however, isolated cases of FG with underlying malignancy of the soft tissue, genitourinary, and gastrointestinal systems have been described. In those cases, it appears that malignancy is overlooked in a significant proportion of patients, resulting in a delayed diagnosis and initiation of cancer treatment.

Methods

We report on a recent case of FG with underlying colorectal cancer and performed a systematic literature review of malignancies presenting as or complicating Fournier’s gangrene over the past ten years, using the National Institutes of Health US National Library of Medicine (PubMed) database. We considered all publications in the English language. Terms used for the search included: “Fournier gangrene,” “Fournier's gangrene,” “Fournier's gangrene,” “Fournier disease,” “Fournier's disease,” “Fournier's disease,” and “necrotizing fasciitis of the perineum and genitalia,” as well as the qualifiers of “malignancy,” “carcinoma,” and “cancer.” Based on these criteria, the database resulted in 157 relevant articles on FG. Twenty case reports and seven case series or review articles included specific information on the relationship or presence of malignancy in FG and these were included in this literature review. A subset of publications focused on necrotizing fasciitis of the thigh, related to soft tissue sarcoma. These were not included in this review.

## Case presentation

A 70-year-old male patient, with a past medical history of dementia, atrial fibrillation, prior myocardial infarction, hypertension, and benign prostatic hyperplasia presented to an outside hospital with fever, scrotal pain, and overlying erythema and fluctuance of the perineum/medial gluteal region.

Computed tomography of the abdomen and pelvis with IV contrast revealed a complex 8.5 × 10.5 × 6.8 cm^3^ right-sided pelvic fluid collection containing gas and surrounding inflammatory changes that extended into the soft tissues of the perineum, gluteal areas, and scrotum.

Under the presumptive diagnosis of FG, the patient was started on broad-spectrum antibiotics, intravenous fluid resuscitation, and underwent emergent surgical debridement. In the operating room, an incision and drainage of the abscess and debridement of gangrenous tissue were performed through a perineal to the right gluteal incision. The patient also received a diverting sigmoid loop colostomy during that hospital stay. No tissue biopsies were obtained.

**Figure 1:**
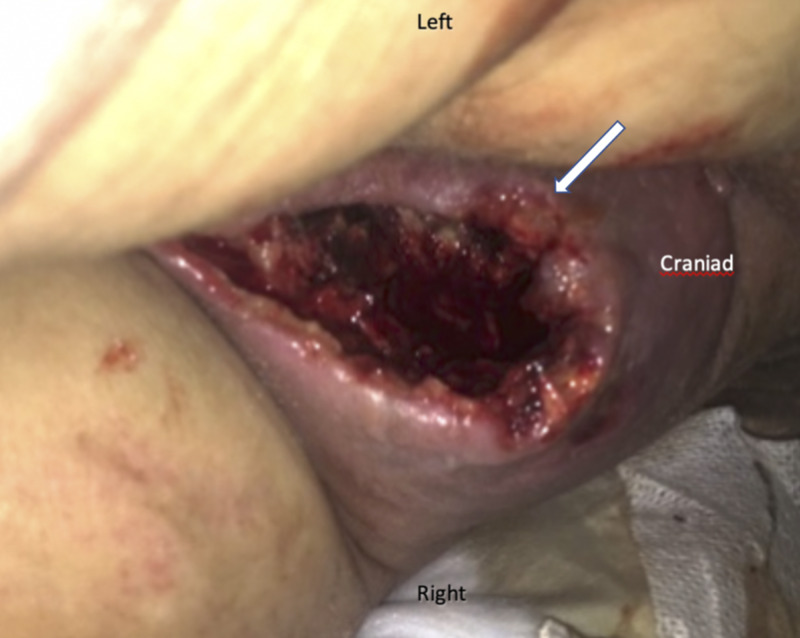
Intraoperative image at re-exploration: necrotizing infection of the right inferior gluteal area (patient in lateral decubitus position)

Two months later the patient returned to the emergency department with a necrotic, foul-smelling chronic perineal/gluteal wound and surrounding skin erythema (Figure 1). Under the diagnosis of recurrent or persistent FG, the patient was treated with broad-spectrum antibiotics, intravenous fluid resuscitation, and underwent emergent surgical re-exploration and debridement.

On exploration, a firm mass was identified in the right retro-rectal space. Biopsy of the lesion revealed partially necrotic granulation tissue containing markedly atypical, pleomorphic cells, with abundant mitotic figures, atypical mitoses, and a sheet-like growth pattern. Immunohistochemistry revealed patchy expression of CD138, weak expression of CD68, and diffuse staining for Oscar keratin, consistent with malignant epithelioid neoplasia, favoring carcinoma.

**Figure 2:**
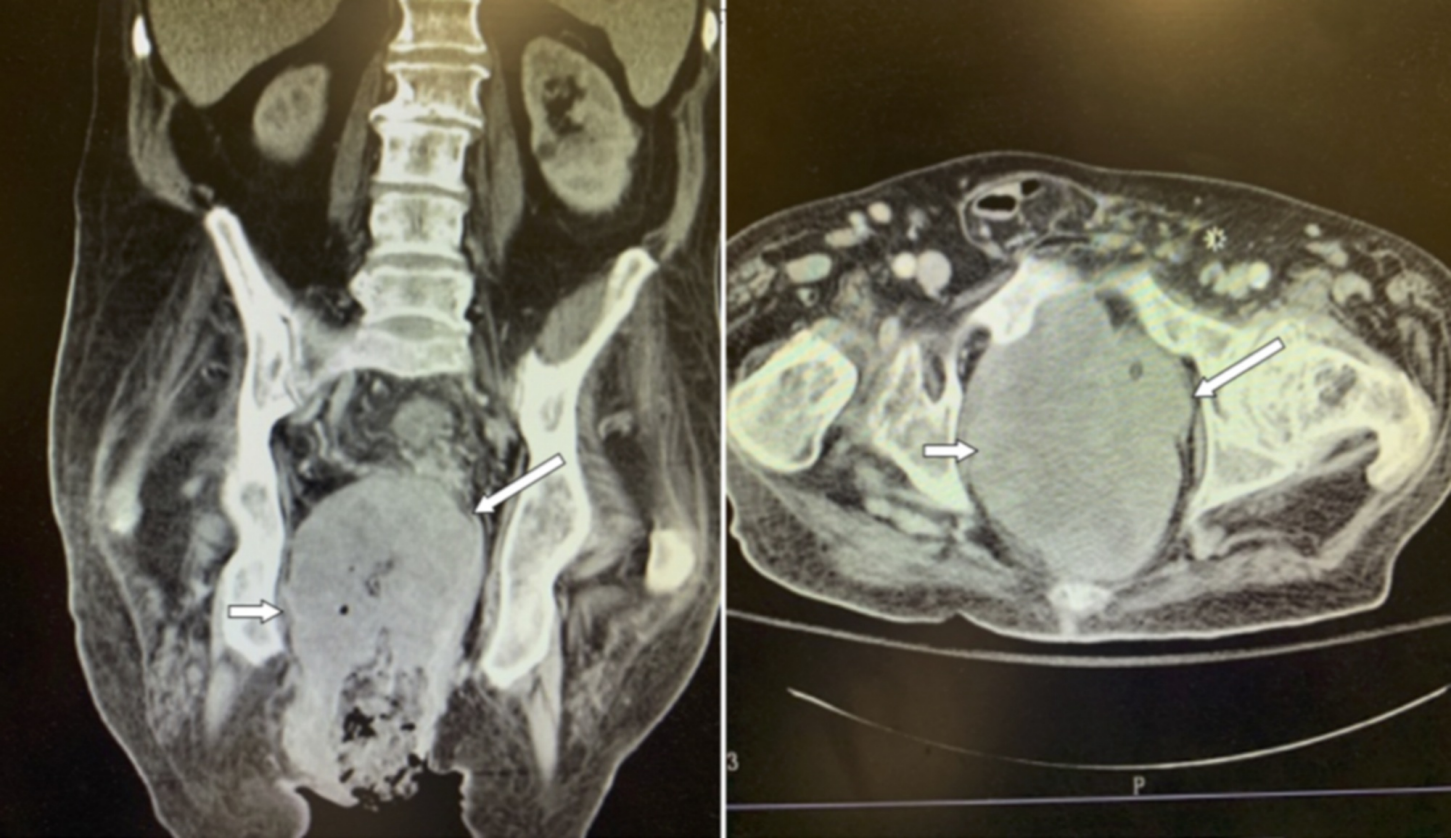
Computed tomography on readmission, showing the complex abscess cavity (long arrow) and mass lesion (short arrow)

Staging computed tomography of the chest, abdomen, and pelvis revealed a 14 × 16 × 10 cm^3^ necrotic pelvic soft tissue mass, surrounding the rectum and anus (Figure 2). There was evidence of multi-focal metastatic disease to the lungs and bones. After extensive discussions, the patient declined further workup and additional local or systemic therapy and elected discharge to hospice care.

## Discussion

Epidemiology and risk factors

In 2009, the overall incidence of FG in the United States was 1.6/100,000 per year in males. FG contributes to 0.02-0.09% of all patients admitted to a hospital. The male-to-female ratio of malignancy-induced FG is 18:2 with the most common age presentation of 50-73 years (range 24-82). This is similar to the FG of other etiology (male-to-female ratio 10:1, age at presentation 50-79 years) [2].

The main risk factors for FG include patients who are diabetic (25-50%), alcohol-dependent (10-60%), elderly, malnourished, or otherwise immunocompromised. Other frequently associated co-morbidities include peripheral vascular disease, chronic hypertension, renal insufficiency, smoking, obesity, intravenous drug use, and immobility (spinal cord injury). Local procedures and chronic indwelling catheters increase the risk of infection. Chemotherapy as a treatment for a previously diagnosed unrelated cancer was seen in 15% (3/20) of the case reports [3-7].

Pathogenesis

As cancer cells replicate, tumors may outgrow their blood supply and infiltrate adjacent tissues. This causes necrosis within the tumor and/or tumor ulceration, which increases susceptibility for microbial infection via hematogenous or direct inoculation. Infections are often polymicrobial and may contain anaerobe pathogens. The infection further promotes local tissue destruction and micro-thrombosis of surrounding blood vessels [8]. Bacteria that possess certain tissue-spreading factors, may spread along with fatty tissue and fascial planes, leading to progressive cellulitis and potentially necrotizing fasciitis [8].

Clinical presentation

Patients with malignancy-induced FG usually present with a clinical picture similar to FG of any other etiology: local (scrotal/perineal/medial thigh) pain and swelling is seen in 85% of patients (17/20); peri-anal pain was most prominent in about 10% of patients (2/20); erythema is seen in 35% (7/20) and skin necrosis in 65% (13/20), but ulceration of the skin is less common (10%, 2/20); purulent discharge is present in 35% (7/20) and crepitus in only 25% (5/20). An obvious mass lesion is detected on physical examination in only 10% of cases (2/20) [5,9-11].

Diagnosis

FG is primarily a clinical diagnosis. A thorough history and physical examination should include information on a recent local or distant cancer diagnosis, specifically focusing on anorectal, urogenital, and skin and soft-tissue malignancies, as well as the more common related causes of FG, including folliculitis, peri-anal anal or scrotal infections, recent hemorrhoidectomy or other anorectal procedures, and the use of indwelling urethral catheters [7,12-14].

In reports of malignancy-related FG, completion of a digital rectal examination (DRE) was recorded in only 30% (6/20) patients. Over 83% (5/6) of patients who did undergo DRE demonstrated positive findings, including a palpable mass (4), purulent anal drainage (1), and pain out of proportion (1). This over 80% rate of positive findings on DRE is consistent with a study by the World Journal of Gastroenterology in 2015 which showed that preoperative DRE resulted in positive findings in 75% of FG with underlying rectal cancer and it highlights the importance of including a thorough anorectal examination in all patients with FG [1,3,15-17].

It is important to note that FG can originate and spread entirely in the deeper soft tissues and anatomic spaces and that the overlying skin may remain intact and relatively free of signs of infection for an extended period of time [9].

As part of their laboratory evaluation, each patient should have a complete blood count, basic metabolic panel, and coagulation profile. In addition, patients with signs of more severe infection or sepsis/shock should have a complete metabolic panel, serum lactate, blood cultures, and other laboratory data (i.e., C-reactive protein, CRP, and procalcitonin) in alignment with standard sepsis guidelines [18]. The Laboratory Risk Indicator Necrotizing Fasciitis (LRINEC) score has been used to facilitate the diagnosis of necrotizing fasciitis [18]. It is included in many medical calculator applications and includes the measurement of CRP, hemoglobin, white blood cell count, serum sodium, creatinine, and blood glucose level. Scores of ≥6 raise the suspicion for necrotizing fasciitis/FG and scores of ≥8 are strongly predictive of the disease [5,17]. Initial studies of the LRINEC score showed a 92% positive predictive value in diagnosing FG but subsequent studies have questioned its utility [19].

Computed tomography of the abdomen pelvis down to the proximal thighs (with oral, IV, and possibly rectal contrast) should be considered for all patients to define the extent of the disease and involvement of distinct anatomic spaces. CT has been proven to have greater specificity than plain film radiography or ultrasonography for identifying the process and the extent of the disease and can help to identify a local or distant malignancy [13]. Common CT findings of FG include soft-tissue stranding and edema, asymmetric fascial thickening, coexisting fluid collections, subcutaneous or deep soft tissue emphysema, or findings consistent with a mass lesion, invading the surrounding tissues [5,6]. In our review, CT was performed in 70% (14/20) of the patients; all patients showed at least one finding, and 85% (12/14) demonstrated a mass lesion with inflammation extending into soft tissue [7,12].

Ultrasonography may be helpful as a rapid initial (bedside) imaging modality and can be useful when CT is not immediately available. Sonographic findings of FG may include identification of fluid collections or mass-like lesion, tissue edema, or air shadows indicative of tissue emphysema [3,5].

Prompt diagnosis of FG is essential, and physicians must have a low threshold for early surgical intervention which significantly improves patient outcomes.

Tissue biopsy

Tissue histopathology is considered the golden standard to confirm the diagnosis of FG and distinguish the disease from other forms of soft tissue infection. Histologic evaluation of the involved tissues reveals widespread necrosis of superficial and deep soft tissues and fascia, fibrinoid microvascular thrombosis of small and intermediate vessels, polymorphic cell infiltration, and necrotic detritus [8,13]. Surgeons should obtain tissue biopsies from various depths of the affected areas in all FG patients during exploration and debridement, to confirm the diagnosis and identify a potentially associated malignancy [14].

In our review, rectal adenocarcinomas were responsible for 70% (14/20) of cases. The remaining cases included two colonic adenocarcinomas and isolated cases of prostatic adenocarcinoma, scrotal pleomorphic sarcoma, urethral squamous cell carcinoma, and scrotal squamous cell carcinoma [3,5,9,10]. In two patients with malignancy-induced FG, the tissue was not sent for histopathologic analysis. The diagnosis and treatment of the underlying neoplasm were delayed for over one year and was associated with worse overall survival [20].

Treatment and prognosis

FG secondary to an underlying malignancy is a rare condition with a high rate of mortality (up to 90%) and remains a challenge to physicians due to its rapid onset and spread of the disease. Prompt diagnosis and aggressive multi-modality treatment have been associated with improved outcomes. The development of sepsis and multiple organ failure are among the most common causes of death. Prognostic predictions such as the Fournier’s Gangrene Severity Index (FGSI) can be used to risk-stratify patients [19]. The index is comprised of body temperature, heart rate, respiratory rate, serum sodium, potassium, creatinine, bicarbonate, hematocrit, and leukocyte count. Zil-E-Ali et al. showed that FGSI scores of >9 were associated with a 75% mortality, while scores of ≤9 corresponded to a 78% probability of survival [19].

The current treatment guidelines include rapid initiation of broad-spectrum antibiotic coverage (after obtaining blood and if possible, tissue cultures), intravenous fluid resuscitation and sepsis/shock management, as well as emergent wide surgical debridement. The antibiotic regimen should cover Staphylococcus, Enterococcus, *Escherichia coli*, and other Gram-negative pathogens and anaerobes (including Bacteroides and Clostridium species). Most commonly used agents include broad-spectrum penicillin with a beta-lactamase inhibitor, a second- or third-generation cephalosporin, a carbapenem, in combination with agents that cover potentially resistant Gram-positive bacteria and anaerobes, i.e., Vancomycin or Daptomycin, Clindamycin, and/or Metronidazole [5,6,14,16]. As soon as culture results are available, a targeted therapy should be implemented. All patients should be closely monitored in an intensive care setting [5,9,12].

At exploration, the surgeon should debride all obviously necrotic tissues, leave the wound open, and plan to re-explore within 24-48 hours (as soon as the patient condition allows) [11,13,19,20]. Tissue sampling for histologic examination and Gram stain/culture should be obtained in all patients. Wound management initially should consist of frequent wet to dry dressing changes and later may include the use of vacuum-assisted wound management systems [5,6].

Hyperbaric oxygen (HBO) therapy has been suggested as an adjuvant therapy to inhibit the growth of anaerobic bacteria in the affected tissues, prevent further extension of tissue necrosis, and assist with wound healing. The use of HBO remains controversial, with recent research failing to identify a clear benefit in all patients [2,10].

FG associated with malignancy carries a mortality risk of at least 20-40%. Poor prognostic indicators include older age (>60 years), the presence of peripheral vascular disease, poor nutritional status, sepsis/shock at presentation, positive blood cultures, and delayed presentation or treatment (>48 hours). The hospital length of stay in these patients ranges from 20 to 130 days [12].

## Conclusions

Malignancy is a potential underlying etiology of FG. The associated cancer diagnosis is initially often missed, leading to a delay in treatment and worse overall outcomes. All patients with FG should have a thorough cancer history, digital rectal examination, appropriate ultrasound or CT imaging, as well as tissue biopsies from multiple sites, to reduce the likelihood of a missed cancer diagnosis. Physicians must maintain a high index of suspicion in patients with FG that do not have common risk factors or where the etiology is unclear. Delay in management of the local malignancy may lead to persistence or recurrence of the infection and significantly worsens overall outcome and survival. 
